# Reviewer acknowledgment

**DOI:** 10.1186/1742-4933-10-13

**Published:** 2013-04-29

**Authors:** Calogero Caruso

**Affiliations:** 1 University of Palermo, Department of Pathobiology and Medical and Forensic Biotechnologies, Corso Tukory 211, Palermo, 90134, Italy

## Abstract

**Contributing reviewers:**

*Immunity & Ageing* would like to thank the following for their assistance with peer review of manuscripts for the journal in 2012.

## 

Arne Akbar

United Kingdom

Giorgio Annoni

Italy

Richard Aspinall

United Kingdom

Carmela Balistreri

Italy

Giovanni Bernardini

Italy

Yang Bian

United States of America

Marcia Blackman

United States of America

Annemieke Boots

Netherlands

Lindsay Brown

Australia

Giuseppe Carruba

Italy

Giuseppina Colonna-Romano

Italy

Francesco Curcio

Italy

Marta Di Carlo

Italy

Mariani Erminia

Italy

Daniela Frasca

United States of America

Tamas Fulop

Canada

Jingang Gui

United States of America

Yingbo He

United States of America

Pernille Hojman

Denmark

Ruth Hubbard

Australia

Dimitris Kletsas

Greece

Anis Larbi

Singapore

Federico Licastro

Italy

Domenico Lio

Italy

Angela Lombardi

Italy

Anastasios Lymperopoulos

United States of America

Philippa Marrack

United States of America

Ilhem Messaoudi

United States of America

Eugenio Mocchegiani

Italy

Roberto Monastero

Italy

Janko Nikolich-Zugich

United States of America

Carlos Orihuela

United States of America

Roberto Paganelli

Italy

Akhilesh Pandey

United States of America

Giuseppe Passarino

Italy

Ananda Prasad

United States of America

Mauro Provinciali

Italy

Annibale Alessandro Puca

Italy

Norman Relkin

United States of America

Gaia Spinetti

Italy

Lishi Sun

United States of America

Kathryn Taylor

United Kingdom

Yuehua Wei

United States of America

Raymond Welsh

United States of America

Jeffrey Woods

United States of America

Zhen Zhao

United States of America

Xiaofei Zhou

United States of America

